# IL-6 and IL-8 secreted by tumour cells impair the function of NK cells via the STAT3 pathway in oesophageal squamous cell carcinoma

**DOI:** 10.1186/s13046-019-1310-0

**Published:** 2019-07-19

**Authors:** Jian Wu, Feng-xia Gao, Chao Wang, Mei Qin, Fei Han, Tao Xu, Zhi Hu, Yang Long, Xue-mei He, Xin Deng, De-lian Ren, Tian-yang Dai

**Affiliations:** 1 ​Department of Thoracic Surgery, The Affiliated Hospital of South West Medical University, Luzhou, Sichuan China; 2 Department of Immunology, Basic Medicine College, South West Medical University, Luzhou, Sichuan China; 3 Experimental Medicine Center, The Affiliated Hospital of South West Medical University, Luzhou, Sichuan China; 4 Drug Discovery Research Center, Southwest Medical University, Luzhou, Sichuan China; 5 Laboratory for Cardiovascular Pharmacology of Department of Pharmacology, The School of Pharmacy, Southwest Medical University, Luzhou, Sichuan China

**Keywords:** Nature killer cell, IL-6, IL-8, STAT3 signalling, Oesophageal squamous cell carcinoma

## Abstract

**Background:**

Recurrence and metastasis are the leading causes of tumour-related death in patients with oesophageal squamous cell carcinoma (ESCC). Tumour-infiltrating natural killer cells (NK cells) display powerful cytotoxicity to tumour cells and play a pivotal role in tumour therapy. However, the phenotype and functional regulation of NK cells in oesophageal squamous cell carcinoma (ESCC) remains largely unknown.

**Methods:**

Single cell suspensions from blood and tissue samples were isolated by physical dissociation and filtering through a 70 μm cell strainer. Flow cytometry was applied to profile the activity and function of NK cells, and an antibody chip experiment was used to identify and quantitate cytokine levels. We studied IL-6 and IL-8 function in primary oesophageal squamous carcinoma and NK cell co-cultures in vitro and by a xenograft tumour model in vivo. Western blotting was used to quantitate STAT3 (signal transducer and activator of transcription 3) and p-STAT3 levels. Finally, we performed an IHC array to analyse IL-6/IL-8 (interleukin 6/interleukin 8) expression in 103 pairs of tumours and matched adjacent tissues of patients with ESCC to elucidate the correlation between IL-6 or IL-8 and clinical characteristics.

**Results:**

The percentages of NK cells in both peripheral blood and tumour tissues from patients with ESCC were significantly increased in comparison with those in the controls and correlated with the clinical characteristics. Furthermore, the decrease in activating receptors and increase in inhibitory receptors on the surface of tumour-infiltrating NK cells was confirmed by flow cytometry. The level of granzyme B, the effector molecule of tumour-infiltrating NK cells, was also decreased. Mechanistically, primary ESCC cells activated the STAT3 signalling pathway on NK cells through IL-6 and IL-8 secretion, leading to the downregulation of activating receptors (NKp30 and NKG2D) on the surface of NK cells. An ex vivo study showed that blockade of STAT3 attenuated the IL-6/IL-8-mediated impairment of NK cell function. Moreover, the expression of IL-6 or IL-8 in tumour tissues was validated by immunohistochemistry to be positively correlated with tumour progression and poor survival, respectively.

**Conclusions:**

Tumour cell-secreted IL-6 and IL-8 impair the activity and function of NK cells via STAT3 signalling and contribute to oesophageal squamous cell carcinoma malignancy.

**Electronic supplementary material:**

The online version of this article (10.1186/s13046-019-1310-0) contains supplementary material, which is available to authorized users.

## Background

Oesophageal squamous cell carcinoma (ESCC) is the sixth most common cancer worldwide with poor survival [1]. Epidemiological studies have shown that most patients with ESCC die from tumour recurrence and metastases, but the underlying mechanism remains to be clarified [2]. Recently, immunotherapy, such as Car-T and PD-1/PD-L1 antibodies, has been applied to tumour therapy with far-reaching impact as a new therapeutic strategy [3, 4]. Large numbers of lymphocytes have been found to infiltrate tumour tissues for immune surveillance, but tumour cells also develop multiple mechanisms to escape immune surveillance [5–8]. Therefore, the identification of distinct mechanisms for immune escape is important for the search for new therapeutic strategies.

Innate immunity is the body’s first line of defence against tumour recurrence and metastasis. Natural killer (NK) cells are a major component of innate immunity. Convincing evidence has revealed that NK cells derived from bone marrow are released into peripheral blood upon maturation [9, 10]. The proportion of NK cells is approximately 5–15% of circulating blood lymphocytes. The classical population of NK cells is defined as the CD3-CD56+ subtype, which can be further divided into CD3-CD56^bright^ and CD3-CD56^dim^ subtypes [11]. Increasing evidence reveals that the latter subtype is dominant in tumour-infiltrating NK cells [12]. NK cells can recognize the target rapidly and release cytotoxic effector molecules without Major Histocompatibility Complex (MHC) restriction [13]. Moreover, NK cells have been utilized for immunotherapy for decades (known as adoptive immunotherapy), but the survival of patients with tumours does not obviously improve [14, 15]. One important reason for this lack of improvement is that the function of tumour-infiltrating NK cells could be impaired by the tumour microenvironment [16].

It has been established that many components in tumour tissues modulate the activity of infiltrating lymphocytes to form an immunosuppressive environment [17, 18]. As the main constituent of tumour tissues, primary tumour cells have been reported to play a key role in the inhibition of infiltrating lymphocytes. For instance, tumour cells can polarize macrophages from M1 to M2 phenotypes [19]. Little is known about the relationship between primary ESCC cells and NK cells. In the current study, we investigated the characteristics of NK cells in patients with ESCC and elucidated the mechanism by which ESCC cells regulate the function of NK cells.

## Materials and methods

### Blood and tissue samples

Fresh peripheral blood was collected from healthy volunteers with an average age of 29 years. Fresh peripheral blood, autologous adjacent tissues, and tumour tissues were obtained from ESCC patients during surgery at the First Affiliated Hospital of Southwest Medical University. None of the patients had received radiotherapy or chemotherapy before sampling, and individuals with autoimmune diseases, infectious diseases, or multi-primary cancer were excluded. According to the tumour-node-metastasis (TNM) classification system published by the International Union Against Cancer (Edition 7), the clinical stages of tumours were determined. The study was approved by the Ethics Committee of the First Affiliated Hospital of Southwest Medical University, and prior written informed consent was obtained from each patient.

### Preparation of cancer tissue single cell suspensions

Fresh tumour and adjacent tissues were used for the isolation of tissue-infiltrating lymphocytes. Paired fresh tumour and adjacent tissue samples were cut into small pieces, washed with 4 °C PBS, ground for an hour with a tissue grinder (Wei Ning biological company), and then filtered through the grinder to obtain single cell suspensions.

### Isolation of cancer tissue lymphocytes

A total of 5 mL of lymphocyte separation solution (Tianjin Haoyang Biology Company) was added to 15 mL centrifuge tubes; 650 g of tissue was centrifuged for 20 min; the second layer of milky white lymphocytes was carefully moved to another 15 mL centrifugation tube (Thermo); and the collected lymphocyte cells were re-suspended.

### Flow cytometric analysis

A total of 10^6^ cells were suspended in 100 μl of PBS, and suitable amounts of fluorescently labelled antibodies were added and incubated at room temperature for 30 min. Cells were analysed by flow cytometry (BD Biosciences), and data were analysed using FlowJo software (TreeStar). The antibodies used for flow cytometry are described in Additional file 1.

### Isolation and purification of primary cells

Fresh ESCC tissues were brought to the laboratory in sterile frozen containers containing 5% iodine volts solution within 2 h. Appropriate amounts of sterile PBS solution were added, and the tissues were soaked for 15 min. Small scissors were used to cut off the necrotic parts of the tissue. The small tissue pieces were digested for an hour in Hank’s enzyme-free dissociation buffer (containing 1 mg/mL type IV collagenase, 1 mg/mL hyaluronidase, and 0.25 mg/mL DNase) at 37 °C for 1 h, and the primary oesophageal squamous carcinoma cells were collected. The cells were grown in RPMI1640 (Welgene) supplemented with 10% FBS.

### Animal experiments with ESCC#1 and ESCC#2 primary cells for oesophageal squamous carcinoma identification

All animal experiments were approved by the Animal Ethical and Experimental Committee of Southwest Medical University. Nude mice were purchased from the ChongQing TengXin company. A total of 10^6^ ESCC#1 or ESCC#2 cells were implanted into the left armpit of nude mice. On the 21st day after implantation, the mice were sacrificed, and the tumours were taken for H&E and IHC staining.

### In vitro co-culture of primary oesophageal squamous carcinoma and NK cells

Peripheral blood NK cells were purified by a Human NK Cell Enrichment Set-DM kit (BD) and then expanded with an NK Cell Robust Expansion kit (Stemery) according to the manufacturer’s instructions. The purity of NK cells was > 90%, and the number of cells was increased 500–1000-fold. The primary oesophageal squamous carcinoma cell supernatant was collected and cultured with NK cells for 72 h. According to each group, anti- IL-8 or anti- IL-6 was added (Additional file 1) or not. Finally, the NK cell surface receptor expression levels, proliferation and cytokine expression levels were detected by flow cytometry.

### Antibody chip experiment

Antibody chip experiments were conducted by Shanghai Youningwei Biotechnology Co. Ltd., Art. No: ARY005B.

### Animal experiments

#### Tumour growth model

KYSE150 cells (10^6^) were subcutaneously injected into the left armpit of male nude mice (5 weeks of age, six mice/group). On the 5th day after injection, NK cells were injected via the tail vein, and NK cell injections were given every 5 days thereafter. Tumours were measured with callipers and calculated with the formula: Volume(mm3) = [width2(mm2) x length(mm)]/2. At day 21, tumours were dissected and weighed.

#### Tumour lung metastasis model

Approximately 5 × 10^5^ KYSE150 cells (Central Laboratory of Southwest Medical University) were injected into nude mice via the tail vein. On the 5th day post-injection, NK cells were injected via the tail vein, and NK cell injections were given every 5 days thereafter. After 4 weeks, the mice were sacrificed. The lungs were fixed in 4% paraformaldehyde and stained with H&E. Lung metastasis was counted and quantified in a random selection of high-power fields. All animal studies were approved by the Medical Experimental Animal Care Commission of Southwest Medical University.

### Immunohistochemistry (IHC)

Formalin-fixed and paraffin-embedded tissue sections with a thickness of 4 mm were dewaxed in xylene and a graded alcohol series, then hydrated and washed in phosphate-buffered saline (PBS). After pretreatment in a microwave oven, endogenous peroxidase was inhibited by 3% hydrogen peroxide in methanol for 20 min, followed by avidin-biotin blocking using a biotin-blocking kit (Dako, Germany). Slides were then incubated with IL-6 or IL-8 antibodies overnight in a humid chamber followed by incubation with rabbit secondary antibodies. The slides were developed with the Dako liquid 3,3′-diaminobenzidine tetrahydrochloride (DAB) + substrate chromogen system.

### Cytotoxicity detection of lactate dehydrogenase (LDH)

A cytotoxicity assay was performed using stimulated NK cells as effector cells. NK cells were suspended at 1 × 10^6^ cells/mL in RPMI1640 medium without phenol red. K562 cells (Central Laboratory of Southwest Medical University) were used as the target cells. K562 cells were suspended at 5 × 10^4^ cells/mL and co-cultured with effector cells (50 μL/well each) for 4 h on a 96-well microplate at 37 °C with 5% CO_2_. The effector to target (E:T) cell ratio was 20:1. A colorimetric-based lactate dehydrogenase (LDH) assay (Cytotoxicity Detection Kit^PLUS^; Switzerland) was used, and cytotoxic activity was calculated according to the manufacturer’s instructions. Cell-mediated cytolysis was converted to percent specific lysis (%SL) using the following formula: %SL = [(experimental LDH release-spontaneous LDH release)/(maximum LDH release-spontaneous LDH release)]× 100.

### Grading and scoring of IHC staining

IL-6 and IL-8 staining scores were evaluated semi-quantitatively and graded for both intensity (absent or weak 1; moderate, 2; strong, 3) and extent (percentage of positive cells: < 25%, 1; 25–50%, 2; > 50%, 3). The intensity and extent scores were multiplied to obtain a comprehensive score of 0–9 for each specimen. Composite scores of 0–3 were defined as negative protein expression, scores of 4–6 were defined as weakly positive expression, and scores of 7–9 were considered strong positive expression.

### Western blot analysis

Total proteins were extracted using RIPA lysis buffer (Beyotime) according to the manufacturer’s protocol. Forty micrograms of total proteins were separated on 10% SDS-PAGE gels. The proteins were transferred to PVDF membranes. The membranes were incubated with primary specific antibodies (Additional files 2 and 3) at 4 overnight and then incubated with HRP-conjugated secondary antibodies (Beyotime) for 1 h, and signals were detected with an electrogenerated chemiluminescence (ECL) detection reagent (Amersham Life Science, Piscataway). Relative target protein expression levels were normalized to GAPDH and visualized using ImageJ software.

### Statistics

GraphPad Prism (GraphPad Software) was used to analyse data and create graphs. Continuous data are represented as the mean ± standard deviation. Univariate analysis of variance was used between groups. The comparison between two groups was performed by using Student’s t-test. A *p*-value less than 0.05 was considered statistically significant. All experiments were repeated at least three times.

## Results

### The percentage of natural killer cells is increased in the peripheral blood and tissues of patients with oesophageal squamous cell carcinoma

**Fig. 1:**
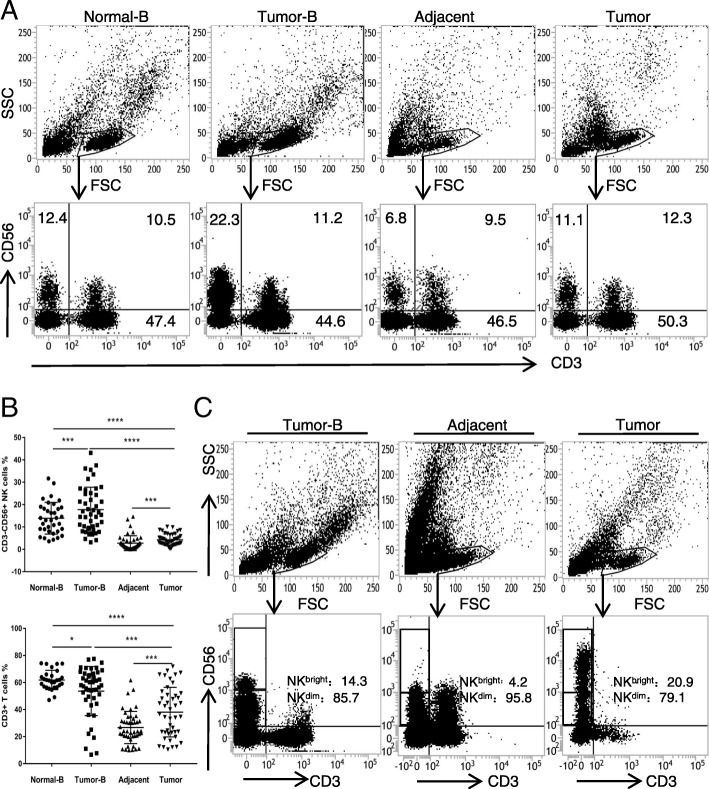
Percentages of NK cells in the peripheral blood of 35 healthy volunteers and in the peripheral blood, tumour and adjacent tissues of 52 ESCC patients. **a** Representative flow cytometry analysis of CD3-CD56+ NK cells in the peripheral blood, tumour and adjacent tissues. Whole peripheral blood-, tumour- and adjacent-tissue-derived cell suspensions were stained with an APC-conjugated antibody (Ab) to human CD56 and a FITC-conjugated Ab to human CD3. CD3–CD56+ NK cell percentages analysed from the lymphocyte gate as defined by the FSC and SSC dot plot. **b** Statistical analysis of CD3–CD56+ NK cell and CD3+ T cell percentages in the peripheral blood, tumour and adjacent tissue. Data analysed by Student’s t-test are shown as the mean ± SEM, *p < 0.05, ***p < 0.001, and ****p < 0.0001. **c** Representative flow cytometry analysis of CD3-CD56^bright^ and CD3-CD56^dim^ NK cells in the peripheral blood and tumour tissues of ESCC patients

To evaluate the potential roles of NK cells in human oesophageal squamous cell carcinoma, we first analysed the percentage of NK cells in the peripheral blood, tumour tissues and matched adjacent tissues of 35 healthy volunteers or 52 patients (Fig. 1a). The characteristics of T cell and NK cell lymphocyte gates were defined by forward (FSC) and side scatter (SSC) dot plots. The results of the flow cytometry analysis revealed that the percentage of CD3-CD56+ NK cells in patient peripheral blood increased significantly compared with that in healthy volunteer peripheral blood, while CD3+ T cells displayed inverse percentages (*p* < 0.05) (Fig. 1b). Moreover, the percentage of NK cells and T cells in ESCC tissues was significantly higher than that in matched adjacent tissues (*p* < 0.05) (Fig. 1b). Convincing evidence has revealed that CD56^dim^ NK cells are mainly distributed in peripheral blood for immune surveillance and that CD56^bright^ NK cells are mainly located in tissues for immunomodulation via cytokine secretion [12]. Intriguingly, we observed that the CD56^dim^ NK cells were the predominant subtype in tumour tissues, which was consistent with the subtype content in peripheral blood but different from that in adjacent tissues (Fig. 1c). This finding suggests that the tumour-infiltrating NK cells may originate in the peripheral blood. Altogether, these results show that the percentage of tumour-infiltrating NK cells is increased in the peripheral blood and tissues of patients with oesophageal squamous cell carcinomas.

### The function of tumour-infiltrating NK cells is impaired in tissues of patients with oesophageal squamous cell carcinoma

**Fig. 2:**
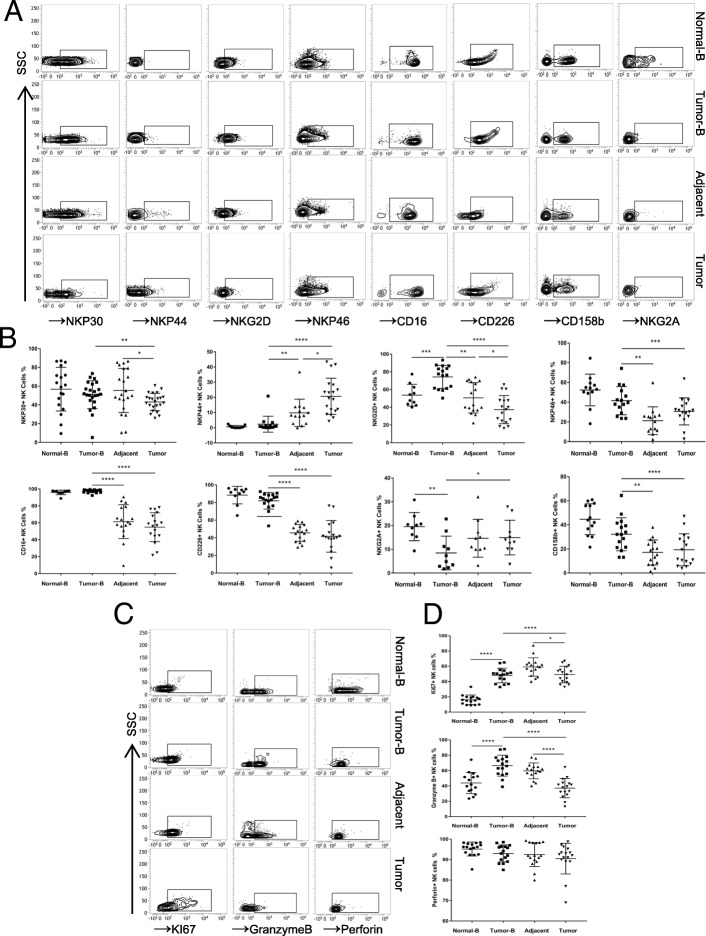
Phenotypic features of NK cells from the peripheral blood of normal volunteers and peripheral blood, tumour and tumour-adjacent tissues of ESCC patients. **a** Peripheral blood-, tumour- and adjacent tissue-derived cell suspensions were stained with Abs to CD3, CD56, CD16, NKp30, NKp44, NKp46, NKG2D, CD226, NKG2A, and CD158b. NK cells were gated as CD3-CD56+ events, and the expression levels of CD16, NKp30, NKp44, NKp46, NKG2D, CD226, NKG2A, and CD158b were then analysed. **b** Symbols represent individual values from 9 to 21 ESCC patients or volunteers analysed individually, *p < 0.05, **p < 0.01, ***p < 0.001, and ****p < 0.0001. Functional characteristics of NK cells in ESCC patients. **c** Representative dot plots of KI67, granzyme B, and perforin expression levels in NK cells from the peripheral blood of normal volunteers and paired patient blood, tumour-adjacent and tumour tissues from each ESCC patient. **d** Statistical analysis of KI67+, granzyme B+, and perforin+ NK cell percentages. Symbols represent individual values from 15 to 16 ESCC patients or volunteers analysed individually. *p < 0.05, **p < 0.01, ***p < 0.001, and ****p < 0.0001. Each experiment was repeated three times

Next, we investigated the phenotype of NK cells in the peripheral blood and tissues. The expression of activating or inhibitory receptors on the surface of NK cells was determined by flow cytometry. In peripheral blood, the level of NKG2D, an activating receptor, was increased, and the level of NKG2A, a known inhibitory receptor, was significantly decreased in patients with ESCC compared with healthy volunteers (*p* < 0.05) (Fig. 2a and b), suggesting that the activity of NK cells was enhanced. In comparison with matched ESCC patient blood samples, lower percentages of activator receptors, such as NKp30, CD16, NKp46, NKG2D, and CD226, and higher percentages of inhibitory receptors, such as NKG2A, were observed in adjacent tissues and tumour tissues (*p* < 0.05) (Fig. 2a and b). Moreover, the expression of the activating receptors NKp30 and NKG2D in tumour tissues was lower than that in adjacent tissues, suggesting that the phenotype of NK cells could be modulated after infiltration into the tumour microenvironment. Next, we detected the expression of effector molecules in tumour-infiltrating NK cells and found that the expression of granzyme B was decreased in comparison with that in adjacent tissues or peripheral blood, but perforin expression was not significantly changed (Fig. 2c and d). In addition, we further detected KI67 expression, which is a marker of cell proliferation. The results revealed that Ki67 expression was significantly decreased in tumour-infiltrating NK cells, suggesting that the proliferation potential of NK cells was impaired. Taken together, these results demonstrate that the phenotype and function of tumour-infiltrating NK cells is impaired in tumour tissues of patients with oesophageal squamous cell carcinoma.

In addition, clinicopathological variables of 52 patients were collected; the percentage of tumour-infiltrating NK cells correlated with the G stage and TMN stage of ESCC. Although there was a certain trend in the correlation, it was not statistically significant due to the insufficient number of samples (Additional files 1, 2 and 3).

### Primary oesophageal squamous carcinoma cells inhibit NK cell function in vitro

**Fig. 3:**
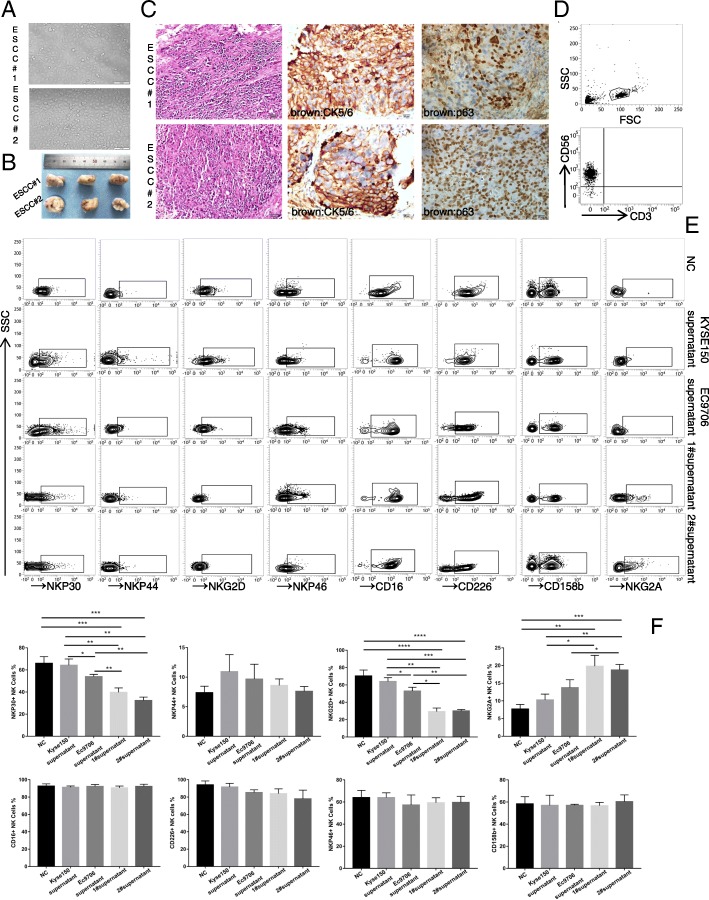
Primary oesophageal squamous carcinoma cell isolation, identification and co-culture. **a** ESCC#1 and ESCC#2 morphology under a light microscope. **b** Representative nude mice with subcutaneous tumours derived from 1 × 10^6^ ESCC#1 or ESCC#2 cells. **c** Representative images of H&E and IHC staining for CK5/6 and P63 in mouse xenograft tumours are shown. Scale bar of H&E staining images indicates 50 μM. Scale bar of IHC staining images indicates 20 μM **d** Isolated and pure NK cells from volunteer peripheral blood obtained by using an NK cell purification kit to achieve approximately 95% NK cell purity with less than 1% T cells. **e** RPMI1640 media (NC), the supernatant of KYSE150, EC9706, ESCC#1 or ESCC#2 cells co-cultured with NK cells. The CD56 + CD3^_^ NK cells were stained with CD16, NKp30, NKp44, NKp46, NKG2D, CD226, NKG2A, and CD158b and analysed by flow cytometry. **f** Statistical analysis of NKp30+, NKG2D+, NKp44+, NKp46+, CD226+, NKG2A+, and CD158b + NK cell percentages

**Fig. 4:**
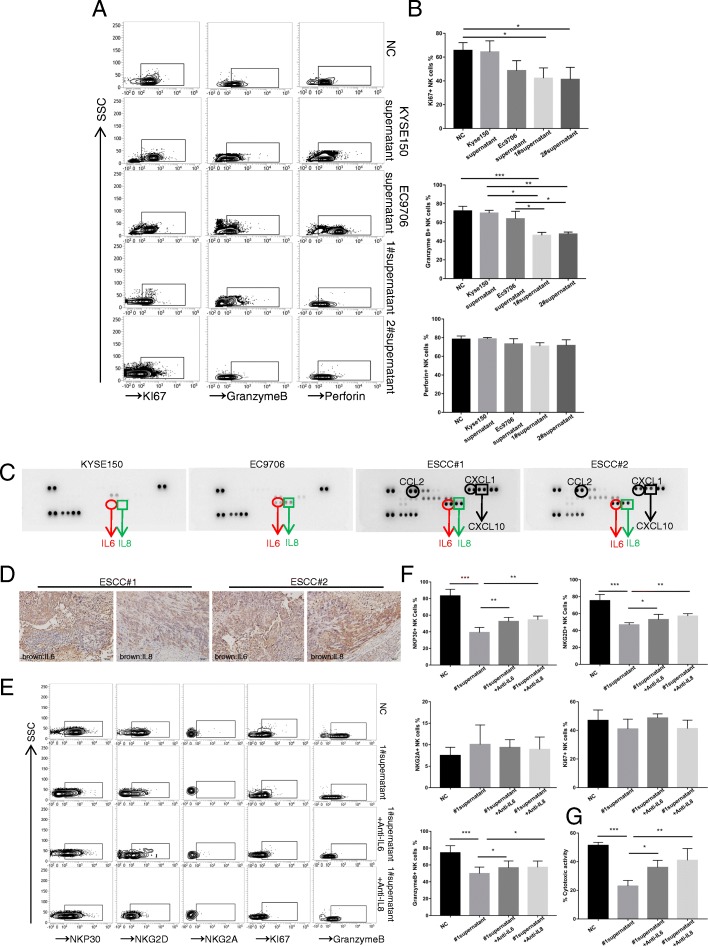
IL-6 and IL-8 inhibit the function of NK cells in vitro. **a** Representative isobaric map of KI67, granzyme B and perforin in the NK cells of five different groups. **b** Statistical analysis of KI67+, granzyme B+ and perforin+ NK cell percentages. *p < 0.05, **p < 0.01, ***p < 0.001, and ****p < 0.0001. **c** Antibody chip assay showing that the levels of IL-6 and IL-8 are highest in the KYSE150, EC9706, ESCC#1 and ESCC#2 cells supernatants. **d** IL-6 and IL-8 expression was assessed in xenograft tumour specimens by IHC. Scale bar of IHC staining images indicates 50 μM. **e** Different conditional media were used to stimulate NK cells for 24 h, and NKp30, NKG2D, NKG2A, KI67, and granzyme B levels were detected by flow cytometry. **f** Statistical analysis of NKp30+, NKG2D+, NKG2A+, KI67+, and granzyme B+ NK cell percentages. *p < 0.05, **p < 0.01, ***p < 0.001, and ****p < 0.0001. **g** An LDH assay was performed to detect NK cell cytotoxicity in four groups. Each experiment was repeated three times

Primary tumour cells are one of the main constituents of tumour tissues and are reported to contribute to immune system suppression. For instance, tumour cells recruit Treg cells to tissues to suppress the activity of infiltrating immune cells or secrete multiple cytokines to sustain the immunosuppressive microenvironment to help tumour cells survive [18]. To uncover the underlying mechanism of the impairment of tumour-infiltrating NK cells in ESCC, we first sought to isolate primary ESCC cells in vitro. Our results showed that two primary cell lines were obtained, namely, ESCC#1 and ESCC#2 (primary cells of oesophageal squamous cell carcinoma#1 and primary cells of oesophageal squamous cell carcinoma#2), the morphology of which was homogeneous under the light microscope. Moreover, the two primary cell lines were both cancerous in tumorigenicity assays in nude mice (Fig. 3a and b). The pathologist confirmed that the two primary cell lines were moderately differentiated squamous cell carcinomas via H&E staining and IHC for CK5/6 and p63 (Fig. 3c). Next, we purified NK cells from peripheral blood and cultured them in vitro for 15 days. After co-culturing the NK cells with the ESCC#1 and ESCC#2 supernatants (Fig. 3d), we investigated whether the NK cell characteristics were altered. The results showed that the levels of the activating receptors NKG2D and NKp30 (Fig. 3e and f) and the levels of the effector molecule granzyme B (Fig. 4a and b) were suppressed in the ESCC#1 and ESCC#2 co-cultured supernatants in comparison with those in the RPMI1640 medium (NC) supernatant of healthy oesophageal cell lines KYSE150 and EC9706. After co-culturing with NK cells, apart from NKp30 and NKG2D, there was no significant difference between the KYSE150 and EC9706 groups in terms of other activating receptors or inhibitory receptors, even though EC9706 is a well-recognized invasive oesophageal squamous cell carcinoma strain. However, compared with that in the KYSE150 and EC9706 groups, the NKG2A expression level in the ESCC#1 and ESCC#2 groups was higher. These results indicate that primary ESCC cells impaired the activity and function of NK cells, and the effect was stronger than that of the cell lines.

### Primary ESCC cells impair the function of NK cells by secreting IL-6 and IL-8 cytokines

Increasing evidence has revealed that tumour cells can modulate immune cells by secreting multiple cytokines. To address whether the primary ESCC cells inhibited the function of NK cells by secreting cytokines, we performed a cytokine array. The results revealed that the levels of five cytokines, IL-6, IL-8, CCL2, CXCL1, and CXCL10, were significantly changed between the cell lines and the primary cell lines (Fig. 4c). In addition, we noticed that the relative expression levels of IL-6 and IL-8 were both upregulated in the EC9706 group compared with those in the KYSE150 group. In addition, through IHC, we confirmed strong positive staining of IL-6 and IL-8 proteins in the xenograft tissues (Fig. 4d). Although numerous papers have reported that IL-6 and IL-8 can enhance the invasion and metastasis of solid tumours, few studies have discussed how IL-6 and IL-8 modulate the function of NK cells [20, 21]. To determine whether the ESCC cells impair the function of NK cells by secreting IL-6 and IL-8, 5 μg of IL-6 and IL-8 antibodies was added to the supernatant of ESCC#1 cells. The ESCC#1 group with added IL-6 and IL-8 antibodies displayed restored expression levels of NKp30, NKG2D and GranB in comparison with the co-culture group (Fig. 4e and f). Moreover, we confirmed that the cytotoxicity of NK cells was impaired after co-culturing with the supernatant of ESCC#1, which could also be restored by the addition of IL-6 or IL-8 antibodies (Fig. 4g).

**Fig. 5:**
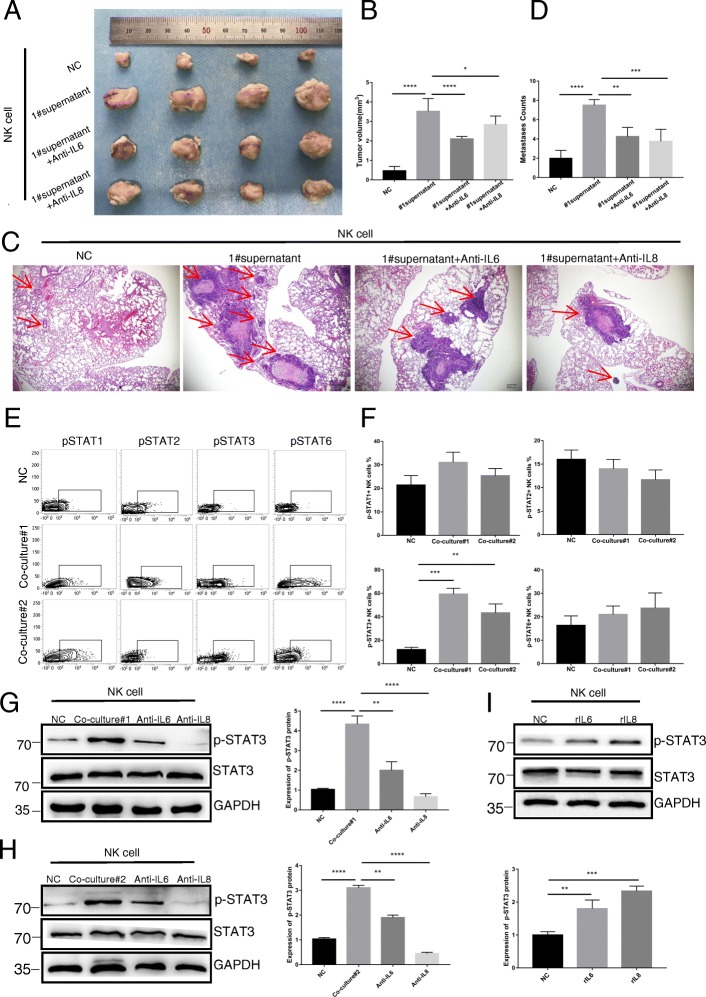
IL-6 and IL-8 inhibit the function of NK cells in vivo. Xenograft model: **a** A total of 1 × 10^6^ ESCC#1 cells were injected into the left armpit of nude mice. After three rounds of NK cell injection, mice were sacrificed, and their tumours were measured. **b** The mean tumour volumes of the four groups are shown. **c** The number and malignancy degree of xenograft tumours was assessed in xenograft tumour specimens by H&E. Scale bar of H&E-stained images indicates 200 μM. **d** Statistical analysis of the number of lung neoplastic foci of four groups of nude mice. IL-6 and IL-8 inhibit NK cell function by increasing STAT3 phosphorylation. **e** Flow cytometry analysis of STAT1, STAT2, STAT3, and STAT6 expression levels in NK cells after three stimulations with different types of NK cell supernatant for 2 h. **f** Statistical analysis of STAT1+, STAT2+, STAT3+, and STAT6 + NK cell percentages. **g** Western blotting was used to detect STAT3 phosphorylation levels in NK cells after co-culture with ESCC#1 supernatant, co-culture with ESCC#1 supernatant+anti-IL-6 supernatant, and co-culture with ESCC#1 supernatant+anti-IL-8 supernatant for 2 h. Statistical analysis of p-STAT3 protein expression levels in NK cells. *p < 0.05, **p < 0.01, ***p < 0.001, and ****p < 0.0001. Each experiment was repeated three times. **h** Western blotting was used to detect STAT3 phosphorylation levels in NK cells after co-culture ESCC#2 supernatant, co-culture ESCC#2 supernatant+anti- IL-6 supernatant, and co-culture ESCC#2 supernatant+anti- IL-8 supernatant stimulation for 2 h. Statistical analysis of p-STAT3 protein expression levels in NK cells. **i** Western blotting was used to detect STAT3 phosphorylation levels in NK cells after RPMI1640 (NC), 100 ng/mL rIL-6 or 100 ng/mL IL-8 stimulation for 2 h. Statistical analysis of p-STAT3 protein levels in NK cells. *p < 0.05, **p < 0.01, ***p < 0.001, and ****p < 0.0001. Each experiment was repeated three times

In vivo, a KYSE150 ESCC cell transplantation model was established in mice with thymic and NK cell defects. Our results revealed that after injecting normal humanized NK cells via the tail vein, the tumour size of BALB/c nude mice was significantly lower than that of mice injected with NK cells co-cultured with ESCC#1 supernatant; furthermore, the tumour size was restored by the addition of IL-6 and IL-8 antibodies (Fig. 5a and b). A similar result was observed in the lung metastasis model, indicating that the impairment of NK cells by ESCC was dependent on IL-6 and IL-8 (Fig. 5c and d). Taken together, these data suggest that ESCC cells impair the function of NK cells through IL-6 and IL-8 secretion.

### IL-6 and IL-8 impair the activity and function of NK cells through the STAT3 signalling pathway

**Fig. 6:**
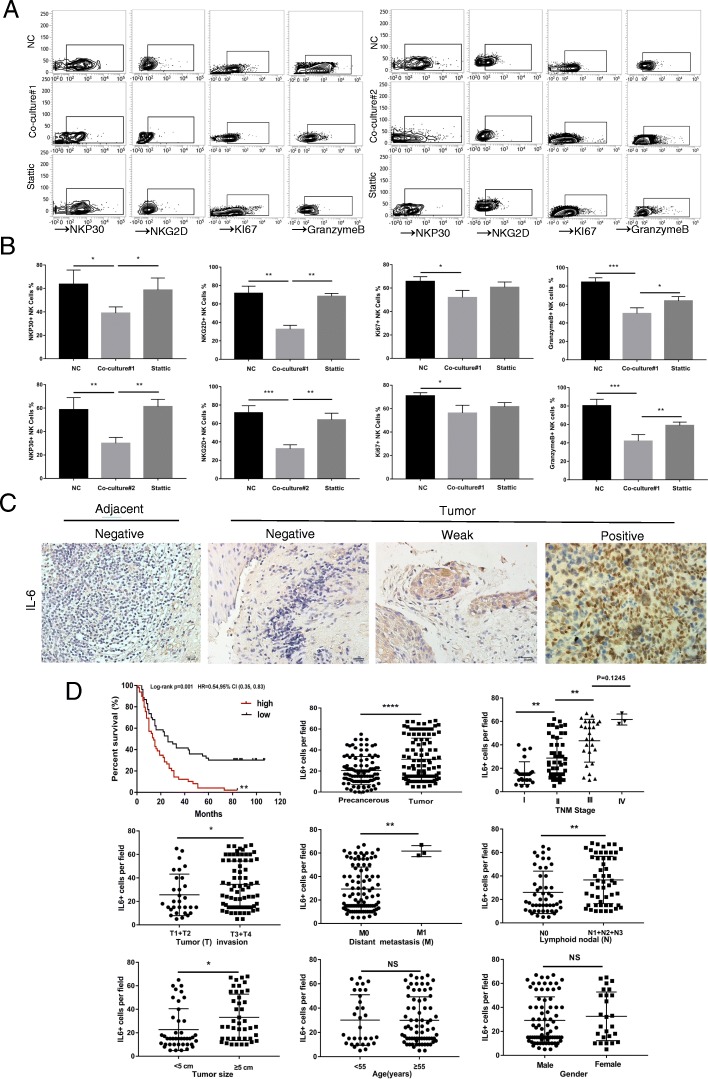
STAT3 feedback assay and correlation between IL-6 and multiple clinical parameters. **a** Flow cytometry was performed to detect the expression levels of NKP30, NKG2D, KI67 and granzyme B in co-culture models stimulated with 10 mM Stattic for 24 h in vitro. **b** Statistical analysis of NKp30+, NKG2D+, KI67+, and granzyme B+ NK cell percentages in three groups. Each experiment was repeated three times. **c** The expression level of IL-6 in paired tumour and adjacent tissues was detected by IHC. Scale bar of IHC staining images indicates 20 μM. **d** The percentage of IL-6-expressing cells was analysed for putative correlations with multiple clinical parameters. For the cumulative survival curves, patients were separated into two groups by the median value of tumour-infiltrating IL-6-positive cell percentages, and Kaplan-Meier plots were used to calculate cumulative survival differences. *p < 0.05, **p < 0.01, and ****p < 0.0001. Each dot represents 1 patient

The STAT signalling pathway plays an important role in the differentiation and maturation of NK cells. For instance, STAT1 can enhance IFN-γ production and the cytotoxicity of NK cells; phosphorylated STAT3 can impair tumour immune surveillance and promote tumour escape from immune control [16]. Increasing evidence has revealed that IL-6 promotes tumour development through the STAT3 signalling pathway [22, 23]. Currently, there are few studies on the relationship between IL-8 and STAT3. To investigate the underlying mechanism of IL-6/IL-8 regulation of NK function, the phosphorylation of STAT1, STAT2, STAT3 or STAT6 was detected in NK cells co-cultured with IL-6 or IL-8 for 2 h. The results showed that the level of p-STAT3 was increased in NK cells, and the level of p-STAT1 was slightly increased (Fig. 5e and f). Furthermore, we detected p-STAT3 levels in NK cells through western blotting and found that the supernatant of primary ESCC cells increased p-STAT3 levels in NK cells, which could be reversed by IL-6 or IL-8 antibody treatment (Fig. 5g and h). In addition, we used 100 ng/mL rIL-6 and rIL-8 to stimulate NK cells for 2 h and then detected p-STAT3 levels in NK cells through western blotting. The results showed that rIL-6 and rIL-8 treatment promoted STAT3 phosphorylation to varying degrees (Fig. 5i). Moreover, we further inhibited the phosphorylation of STAT3 using Stattic, an inhibitor of STAT3, and found that the phenotype and function of NK cells was restored (Fig. 6a and b). Taken together, these results suggest that the impairment of NK cells by primary ESCC cells is mainly dependent on IL-6- or IL-8-induced activation of STAT3 signalling.

### Both IL-6 and IL-8 correlate with multiple clinical parameters

**Fig. 7:**
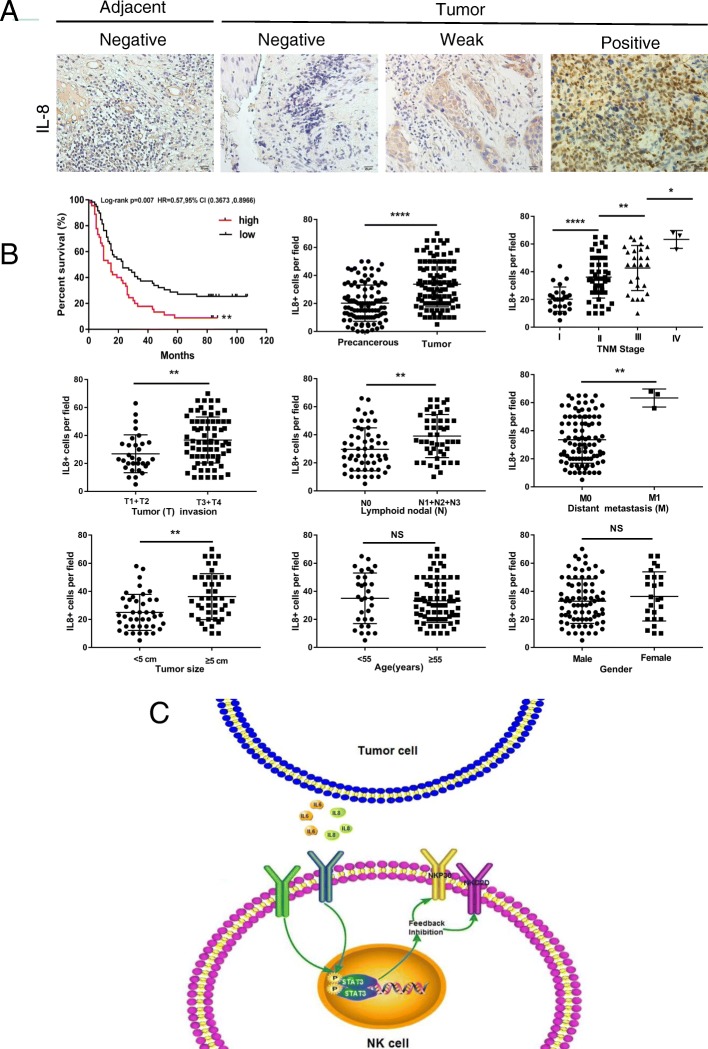
Correlation between IL-8 and multiple clinical parameters. The mechanistic action of IL-6 or IL-8 impairs NK cell function. **a** The expression level of IL-8 in paired tumour and adjacent tissues was detected by IHC. Scale bar of IHC staining images indicates 20 μM. **b** The percentage of tumour-infiltrating IL-8+ cells correlates with multiple clinical parameters of ESCC. The percentage of IL-8+ cells was analysed for putative correlations with multiple clinical parameters. For the cumulative survival curves, patients were separated into two groups by the median value of tumour-infiltrating IL-8+ cell percentages, and Kaplan-Meier plots were used to calculate cumulative survival differences. *p < 0.05, **p < 0.01, and ****p < 0.0001. Each dot represents 1 patient. **c** Schematic diagram. Tumour-derived IL-6 or IL-8 acts on NK cells in an autocrine manner. IL-6 and IL-8 reduce NKp30 and NKG2D expression on NK cell surfaces by enhancing STAT3 phosphorylation, which enhances tumour malignancy and impairs NK cell function

To elucidate the correlation between IL-6 or IL-8 and clinical characteristics, we performed an IHC array to analyse IL-6/IL-8 expression in 103 pairs of tumours and matched adjacent tissues of patients with ESCC (Figs. 6c, d and 7a, b). We found that the number of IL-6- and IL-8-positive tumour cells was significantly increased in tumour tissues in comparison with matched adjacent tissues. Moreover, the number of IL-6- and IL-8-positive tumour cells positively correlated with TNM stage, and high IL-6 and IL-8 expression was found to indicate poor overall survival of ESCC patients when the medium value of all IL-6 and IL-8 percentages was used as a comparison point. In addition, the number of IL-6- and IL-8-positive tumour cells positively correlated with other advanced-stage clinical characteristics, including tumour invasion, lymphoid node and distant metastases and tumour size. IL-6 and IL-8 expression was not correlated with age or gender. Taken together, these results suggest that increased IL-6 and IL-8 expression is involved in ESCC progression and indicates a poor overall survival of patients with ESCC.

## Discussion

Deciphering novel mechanisms of immune suppression within the tumour microenvironment is essential for understanding tumour immune escape. Although many endeavours have focused on identifying the functions of innate immune cells in ESCC, how tumour cells escape from surveillance by innate immune cells remains unclear [24–26]. In the current study, we found that the proportion of NK cells in patients with ESCC increased significantly, and NK activity and cytotoxicity were impaired once the cells had infiltrated the tumour tissues. Further investigation revealed that primary oesophageal squamous cells impaired the function of NK cells via IL-6/IL-8 secretion, which suppressed NKp30, NKG2D and Ki67 expression through the STAT3 signalling pathway. Moreover, IL-6 and IL-8 expression was negatively correlated with poor overall survival in ESCC patients.

Increasing evidence has revealed that the percentage of NK cells in peripheral blood and tumour tissues is increased in ESCC patients and negatively correlated with the survival of patients [27, 28]. For instance. In our study, we found that the percentage of NK cells was increased in the peripheral blood of patients with ESCC in comparison with that in the blood of healthy volunteers. The same results were obtained in the comparison between adjacent and tumour tissues. Consistent with a previous study, we found that the NK cell percentages correlated with the development of ESCC, such as lymph node metastasis and TNM stages. According to the expression of CD56 on the cell surface, NK cells can be divided into two subtypes, namely, CD56^bright^ and CD56^dim^. Convincing evidence has revealed that CD56^bright^ NK cells mainly regulate immune activity via cytokine secretion and that CD56^dim^ NK cells oversee the identification and elimination of abnormal cells [29, 30]. CD56^dim^ NK cells are mainly distributed in peripheral blood, but CD56^bright^ NK cells are located in tissues [31]. In the current study, intriguingly, we found that CD56^bright^ NK cells were abundant in adjacent tissues but rare in tumour tissues. CD56^dim^ NK cells were the predominant NK type in tumour tissues, indicating that the infiltrating NK cells in tumour tissues might be recruited from the peripheral blood.

The activity of NK cells has been proven to be dependent on the expression of multiple activating or inhibitory receptors [32, 33]. For instance, CD16 enhances the function of NK cells by mediating ADCC (antibody-dependent cell-mediated cytotoxicity) activity [34, 35]; NKG2D, an activating receptor, triggers granule release and cytokine production once engaged by ligands [36]. In addition, many other receptors, such as NKp30, NKp44, NKp46, CD226, CD158b and NKG2A, are involved in NK activity regulation [37–42]. In our study, we observed that NKp30 and NKG2D expression in tumour-infiltrating NK cells was downregulated in comparison with that in adjacent tissue, indicating that the activity of NK cells was impaired. Furthermore, we found that granzyme B expression in tumour-infiltrating NK cells was decreased in comparison with that in adjacent tissues and peripheral blood, indicating impaired cytotoxicity. Moreover, we validated that the proliferation potential of tumour-infiltrating NK cells was suppressed by detecting Ki67. In contrast, our results showed that the activity, cytotoxicity and proliferation potential of NK cells was increased in the peripheral blood of patients with ESCC. These data indicate that the function of NK cells was impaired once they had infiltrated tumour tissues.

Convincing evidence has revealed that the tumour tissue environment consists of heterogeneous components, such as tumour cells, fibroblasts, and immune cells [43–46]. The interactions between these cells define the microenvironment of tumour cells and are involved in the growth and metastasis of tumours [47]. Primary tumour cells have been proven to be a pivotal regulator of microenvironment remodelling [48]. For instance, tumour cells induce M1 macrophages to differentiate into M2 macrophages, which promote tumour proliferation and metastasis [49], and TGF-β1 derived from tumour cells suppresses the activity of immune cells [50]. Our results show that the primary tumour cells secreted abundant amounts of IL-6 and IL-8. Several papers have reported that IL-6 inhibits the immune response to tumour cells. For instance, IL-6 inhibits the anticancer response by dampening the IL27/STAT1 signalling pathway [51]. IL-8 is mainly responsible for the recruitment of neutrophils, basophils and T cells [52, 53]. Moreover, accumulated evidence has revealed that IL-8 promotes angiogenesis and metastasis in tumour cells [54]. Currently, little is known about the impact of IL-6/IL-8 on NK cells. In the current study, we found that primary ESCC cells inhibited the expression of NKp30, NKG2D and granzyme B in NK cells through IL-6 and IL-8 secretion in vitro and in vivo.

It has been established that IL-6 and IL-8 are pleiotropic cytokines involved in processes in the inflammatory microenvironment, such as proinflammatory cell chemotaxis, angiogenesis, mitosis and the induction of proliferation [55]. The Janus kinase (JAK)-signal transducer and activator of transcription (STAT) signalling cascade is an important pathway triggered by IL-6 and IL-8 [56]. STAT family proteins include six members that exert different functions. For instance, STAT1 promotes the cytotoxicity of NK cells by increasing IFN-γ expression; the activation of STAT6 has been reported to drive IL-5 and IL-13 production in cultured NK cells and to limit cytotoxic responses [56]. Here, we confirmed through flow cytometry or western blot that the level of p-STAT3 in NK cells increased after IL-6 or IL-8 stimulation. Additionally, the addition of IL-6 or IL-8 antibodies restored the p-STAT3 level. Further studies revealed that a STAT3 inhibitor could also restore the expression of granzyme B, NKG2D and NKp30. It has been reported that STAT3 directly binds to the promoter region of granzyme B [56]. Whether STAT3 acts by binding directly to NKp30 and NKG2D or by some other mechanism requires further study. These data suggest that IL-6 and IL-8 promote STAT3 phosphorylation to impair NK cell function, indicating a new pathway of tumour immune escape during ESCC progression. Most importantly, our findings also shed light on the clinical relevance of IL-6 and IL-8 in ESCC patients. High levels of IL-6 or IL-8 in ESCC tumours correlated with advanced tumour progression and poor patient survival. Since the clinical outcome for ESCC patients remains poor, and few prognostic factors currently exist for this disease following surgery, IL-6 or IL-8 might serve as clinical markers for ESCC patients in the future.

In summary, we identified a novel mechanism by which IL-6 or IL-8 secreted by primary ESCC cells impairs the function of NK cells via the STAT3 signalling pathway. Thus, immune-boosting therapeutic strategies aimed at IL-8 or IL-6 may prove beneficial in ESCC patients.

## Conclusions

In summary, this study identified a novel mechanism by which IL-6 or IL-8 secreted by primary ESCC cells impairs the activity and function of NK cells via the STAT3 signalling pathway. The expression of IL-6 or IL-8 in tumour tissues positively correlates with tumour progression and poor survival. Thus, immune-boosting therapeutic strategies aimed at IL-8 or IL-6 may prove beneficial in ESCC patients.

## Additional files

Additional file 1: Clinical characterization of patients with ESCC. (DOC 26 kb)

Additional file 2: The percentage of tumor-infiltrating NK cells correlated with multiple clinical parameters of patients with ESCC. Percentages of tumor-infiltrating CD3-CD56+ NK cells were analyzed for putative correlations with multiple clinical parameters.*P < 0.05; Each dot represents 1 patient. (PDF 740 kb)

Additional file 3: Different conditional media were used to stimulate NK cells for 24 h and IL-6R and IL-8R were detected by RT-qPCR. IL6-R and IL-8R expression were both upregulated in ESCC#1 and ESCC#2 supernatant group compared to the control.*p < 0.05, **p < 0.01, ***p < 0.001, and ****p < 0.0001. (PDF 211 kb)

## Data Availability

Please contact the corresponding author for all data requests.

## Abbreviations

ESCC: Oesophageal squamous cell carcinomas
ESCC#1: Primary cells of oesophageal squamous cell carcinomas
ESCC#2: Primary cells of oesophageal squamous cell carcinomas
IL-6: Interleukin-6
IL-8: Interleukin-8
MHC: Major Histocompatibility Complex
NK Cell: Natural killer
STAT3: Signal transducersand activators of transcription 3
