# Supplementary material for: IL-6 and IL-8 secreted by tumour cells impair the function of NK cells via the STAT3 pathway in oesophageal squamous cell carcinoma
Source: J Exp Clin Cancer Res. 2019 Jul 19;38:321. doi: 10.1186/s13046-019-1310-0 (PMC6642486; doi:10.1186/s13046-019-1310-0)
Supplement: Supplementary file 1 — Clinical characterization of patients with ESCC. (DOC 26 kb) [file 13046_2019_1310_MOESM1_ESM.doc]

Additional file 1

| Variables | No. of patients |
| --- | --- |
| Sex (male/female) | 48/4 |
| Age (y), median (range) | 61,42-80 |
| G stage(Gx/G1/G2/G3) | 6/22/20/4 |
| Tumor (T) invasion(T1/T2/T3/T4) | 1/21/28/2 |
| Lymphoid nodal(N0/N1/N2/N3) | 26/16/9/1 |
| Distant metastasis(M0/M1) | 51/1 |
| TNM stage(Ⅰ/Ⅱ/Ⅲ/Ⅳ) | 17/8/24/3 |

| Variables | No. of healthy volunteers |
| --- | --- |
| Sex (male/female) | 24/11 |
| Age (y), median (range) | 59.99,45-65 |
| diabetes, heart, cerebrovascular system, immune system and other diseases | no |
| Blood and urine routine, normal liver and kidney function | no |
| Virus, bacterial infection and other diseases | no |

| Variables | Tumor patients Healthy controls  (n=52) (n=35) | *P* |
| --- | --- | --- |
| Age (years) | 61.00 ± 3. 63 59.99 ± 2.80 | 0.146 |
| Sex (Male/Female) | 34 /18 24/11 | 0.542 |
