# Supplementary figures and images for: IL-6 and IL-8 secreted by tumour cells impair the function of NK cells via the STAT3 pathway in oesophageal squamous cell carcinoma
Source: J Exp Clin Cancer Res. 2019 Jul 19;38:321. doi: 10.1186/s13046-019-1310-0 (PMC6642486; doi:10.1186/s13046-019-1310-0)

S2

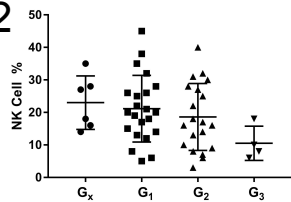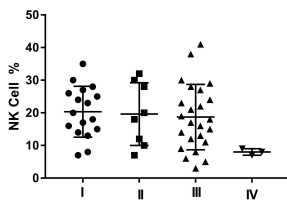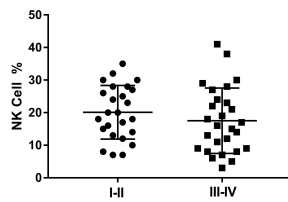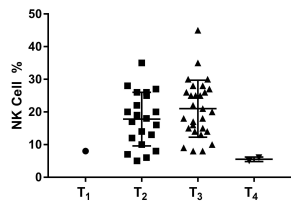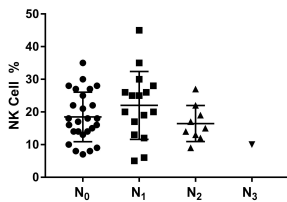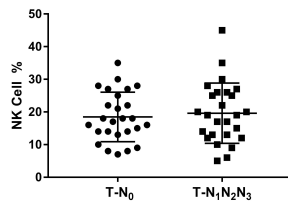

Supplement: Supplementary file 2 — The percentage of tumor-infiltrating NK cells correlated with multiple clinical parameters of patients with ESCC. Percentages of tumor-infiltrating CD3-CD56+ NK cells were analyzed for putative correlations with multiple clinical parameters.*P < 0.05; Each dot represents 1 patient. (PDF 740 kb) [file 13046_2019_1310_MOESM2_ESM.pdf]
