# Supplementary material for: IL-6 and IL-8 secreted by tumour cells impair the function of NK cells via the STAT3 pathway in oesophageal squamous cell carcinoma
Source: J Exp Clin Cancer Res. 2019 Jul 19;38:321. doi: 10.1186/s13046-019-1310-0 (PMC6642486; doi:10.1186/s13046-019-1310-0)
Supplement: Supplementary file 3 — Different conditional media were used to stimulate NK cells for 24 h and IL-6R and IL-8R were detected by RT-qPCR. IL6-R and IL-8R expression were both upregulated in ESCC#1 and ESCC#2 supernatant group compared to the control.*p < 0.05, **p < 0.01, ***p < 0.001, and ****p < 0.0001. (PDF 211 kb) [file 13046_2019_1310_MOESM3_ESM.pdf]

S2 Expression of IL6-R and IL8-R on NK cells via RT-qPCR

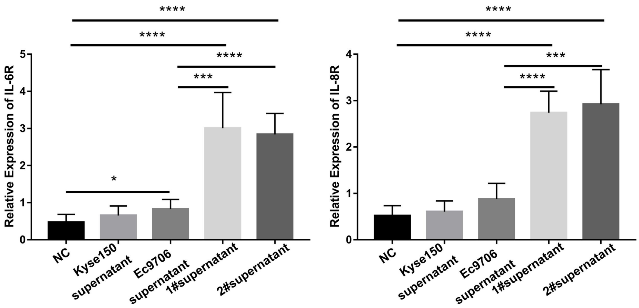

Reverse transcription-quantitative polymerase chain reaction (RT-qPCR). RT-qPCR assays IL-6R and IL-8R expression were performed using a PrimeScript RT reagent kit, SYBR-Green Real-time PCR Master Mix and Permix Ex Taq (both from Takara, Dalian, China), respectively, according to the manufacturers'instructions. The parameters for PCR were as follows: Incubation of the templates at 94˚C for 2 min, followed by 40 cycles of 94˚C for 20 sec, 60˚C for 1 min and 72˚C for 20 sec, and finally incubation at 72˚C for 2 min. We used GAPDH as the housekeeping genes for calculating the relative expression of IL-8R and IL-6R mRNA, respectively. The primers used were as follows: IL-6R forward, 5'-ATGCTGGCCGTCGGCTGC-3' and reverse, 5'- TCAGAGCCCGCAGCTTCC-3'; IL-8R forward, 5'-ATGGAAGATTTTAAC ATGGAGAGTGAC-3' and reverse, 5'-TTAGAGAGTAGTGGAAGTGTGCC-3'; GAPDH forward,5'-CGGAGTCAACGGATTTGGTCGTAT-3' and reverse, 5'-AGCCTTCTCCATGGTGGTGAAGAC-3'.The 2-ΔΔCq method was used to evaluate the relative expression levels of the indicated genes.
